# Restraint stress induced anxiety and sleep in mice

**DOI:** 10.3389/fpsyt.2023.1090420

**Published:** 2023-04-12

**Authors:** Yong-Xia Xu, Guo-Ying Liu, Zhang-Zhang Ji, Yue-Yun Li, Yan-Li Wang, Xue-Yan Wu, Jun-Lin Liu, Dan-Xia Ma, Ming-Kui Zhong, Chao-Bing Gao, Qi Xu

**Affiliations:** ^1^Department of Geriatric Endocrinology, Anhui Geriatric Institute, The First Affiliated Hospital of Anhui Medical University, Hefei, Anhui, China; ^2^Department of Physiology, School of Basic Medical Sciences, Anhui Medical University, Hefei, Anhui, China; ^3^Department of Stomatology, The Second Affiliated Hospital of Anhui Medical University, Hefei, China; ^4^Department of Otorhinolaryngology Head and Neck Surgery, The First Affiliated Hospital of Anhui Medical University, Hefei, Anhui, China; ^5^Department of Human Anatomy, School of Basic Medical Sciences, Anhui Medical University, Hefei, Anhui, China; ^6^School of Mental Health and Psychological Sciences, Anhui Medical University, Hefei, Anhui, China

**Keywords:** anxiety, mouse, sleep disorder, restraint stress, parabrachial nucleus

## Abstract

In humans and animals, exposure to changes in internal or external environments causes acute stress, which changes sleep and enhances neurochemical, neuroendocrine, and sympathetic activities. Repeated stress responses play an essential role in the pathogenesis of psychiatric diseases and sleep disorders. However, the underlying mechanism of sleep changes and anxiety disorders in response to acute stress is not well established. In the current study, the effects of restraint stress (RS) on anxiety and sleep–wake cycles in mice were investigated. We found that after RS, the mice showed anxiety-like behavior after RS manipulation and increased the amounts of both non-rapid eye movement (NREM) and rapid eye movement (REM) sleep in the dark period. The increase in sleep time was mainly due to the increased number of episodes of NREM and REM sleep during the dark period. In addition, the mice showed an elevation of the EEG power spectrum of both NREM and REM sleep 2 h after RS manipulation. There was a significant reduction in the EEG power spectrum of both NREM and REM sleep during the darkperiod in the RS condition. The expression of the c-Fos protein was significantly increased in the parabrachial nucleus, bed nucleus of the stria terminalis, central amygdala, and paraventricular hypothalamus by RS manipulation. Altogether, the findings from the present study indicated that neural circuits from the parabrachial nucleus might regulate anxiety and sleep responses to acute stress, and suggest a potential therapeutic target for RS induced anxiety and sleep alterations.

## Introduction

Since the COVID-19 pandemic, the prevalence of anxiety and depression has increased, which increases the risk of a wide range of diseases and conditions, including sleep disturbances and cognitive dysfunctions (1–3). In patients with anxiety, sleep disturbance is the most common complaint, with insufficient sleep time, poor sleep quality, or alterations of sleep structure (4). In China, the lifetime prevalence of anxiety disorders is up to 7.6%, and the weighted prevalence of anxiety disorders is 5.0% (5), which indicates the need for innovative approaches to address these mental disorders. However, insomnia with comorbid anxiety is a major obstacle to improving clinical outcomes for treatment. Understanding the risk factors for sleep disorders could benefit the intervention of sleep and the prevention of psychiatric disorders.

A high level of stress is associated with an increased risk of developing several pathologies, including sleep disorders and psychiatric diseases (6, 7). More than 60% of patients with generalized anxiety disorder have insomnia symptoms, with increased wakefulness, reduced sleep amount, and less slow-wave sleep (8, 9). A 3 year follow-up of a community-based study showed that the subjects developed insomnia 1 year later, and the sleep response to stressor exposure was significantly associated with their anxiety symptoms (10). In a group of first-year training physicians, poor sleepers sleep less and tend to experience anxiety when exposed to stressors (11). These studies supported the idea that high levels of stress can lead to insomnia and mental disorders in humans. The effect of stress on emotion and sleep has been reported in many experimental studies in rodents. Restraint stress (RS) induces anxiety-like behavior in both mice and rats, with a dysfunction of the amygdala (12–14). In rats, acute RS has been shown to induce increased of wakefulness for 1 h following the RS session, and then rapid eye movement (REM) sleep and non-REM (NREM) sleep rebound (15, 16). Furthermore, 5 days of 30 min RS increased wakefulness and decreased NREM sleep in rats during the light phase (17). In a chronic stress procedure, mice that suffered from 3 weeks of daily water immersion and RS showed an increased NREM sleep time with low power density of delta band, an increased REM sleep time during the dark period in the first week, and only an increase of REM sleep time during the dark phase in the second and third weeks (18). Social defeat stress has a similar effect. Exposing mice to social stress or foot-shock stress results in increased wakefulness in the initial phase, followed by an altered sleep architecture, including prolonged NREM sleep or REM sleep (19–21). These results indicate that stressors are pathological factors that increase the risk of disturbing sleep and emotion, which may increase the development of dementia and metabolic and cardiovascular diseases (22, 23). Nevertheless, the role and potential regulatory mechanisms of stress-induced mood and sleep alterations have not been fully elucidated.

In the present study, mice received RS for 2 h for 3 consecutive days, and an open field test (OFT) and elevated plus maze (EPM) were performed to assess their anxiety-like behaviors. The sleep–wake recording was used to analyze sleep alteration after the RS treatment. Lastly, immunohistochemistry staining was carried out to estimate the neural activity induced by RS. This study attempted to reveal the effects of RS on anxiety and sleep architecture and the potential neural mechanisms in mice.

## Materials and methods

### Animals

The Animal Care and Use Committee of Anhui Medical University approved all animal experiments, and all experimental procedures were carried out in accordance with the Anhui Medical University Animal Experiment Guide, as previously described (23, 24). The C57BL/6 male mice (8–10 weeks old) required for the experiment were purchased from the Anhui Provincial Laboratory Animal Center, China (Certificate No. SCXK [WAN] 2022-001). We housed all mice individually in cages and maintained them in the following controlled environment: 22 ± 1°C,55 ± 5% humidity, 100 lux light intensity, 12-h light/dark cycle (Zeitgeber time, ZT 0: beginning of light period; ZT 12: beginning of dark period. Rodents’ chow and water were available *ad libitum*.

### EEG/EMG electrodes implantation

The procedure for EEG/EMG electrodes implantation was as previously described (23, 25). In brief, all mice were anesthetized with isoflurane and fixed on a stereotaxic apparatus (68,043, RWD Life Science, Shenzhen, China), and two stainless steel pins for EEG recording were inserted into the skulls of the right frontal cortex and the right parietal cortex. The EMG electrodes were inserted into the trapezius muscle on both sides. The electrode base was then fixed on the mouse skull with dental cement. Thereafter, the mice recovered for at least 14 days in their home cages.

### Restraint stress procedure

The mice used for the behavioral test received the following treatment: the mice in the RS group were put into polypropylene centrifuge tubes with multiple air holes for 2 h for three consecutive days, starting at 1 h after the lights on and lasting for 2 h (ZT 1–3) every day. Similarly, mice in the sham group received gentle handling treatment at ZT 1, similar to the RS mice, and had no access to water and food for 2 h, modified from previous reports (26, 27). All mice used for sleep recording were recovered from EEG/EMG electrode implantation and were placed in a sleep recording chamber for 3 days to acclimate. Sleep recording was started, and the EEG/EMG signals during the first 24 h were used as the data under the baseline condition. During this period, the mice have free access to water and food. The mice received RS manipulation, as described early during the sleep recording. The sleep recording was conducted continuously after the end of the RS protocol for 24 h as data for the post-RS (PRS) condition.

### Open field test

The OFT was used to evaluate anxiety-like behaviors and locomotion in mice (28, 29). In a quiet environment, the mouse was placed in the center of the bottom surface of the open field reaction box (50 cm^3^ × 50 cm^3^ × 45 cm^3^), video recording and timing were performed at the same time, and the mouse was allowed to move freely in the box for 5 min. The bottom plane of the open field reaction chamber is divided into nine areas, including the center, perimeter, and corners. The time that the mice passed through each area within 5 min was then recorded and analyzed using a video-tracking system (Shanghai Xinruan Information Technology Co. Ltd., China).

### Elevated plus maze test

The EPM test is another specific experimental paradigm for examining the state of anxiety in mice (30, 31). The EPM is composed of a pair of opposite open arms (35 cm × 5 cm) and a pair of opposite closed arms (35 cm × 5 cm × 15 cm) and a central area, which cross each other in a cross shape. At the beginning of the experiment, one mouse was placed in the central area, while videography and recording were started, and it was allowed to explore freely for 5 min. The video-tracking system was used to analyze the number of times the mouse entered the open arm and the closed arm and the residence time within the 5 min.

### Sleep–wake cycle recording and scoring

After 14 days of recovery from electrode implantation, the mouse was placed individually in a sleep recording chamber for 3 days to acclimatize before starting to record sleep. The EEG and EMG signals were amplified, filtered, acquired, and digitized at a sampling rate of 128 Hz, and recorded using Vitalrecorder software (Kissei Comtec Co., Ltd., Japan). After the recording was completed, SleepSign software (Kissei Comtec Co., Ltd., Japan) was used to parse the EEG/EMG signals into NREM sleep, REM sleep, and wakefulness phases using an epoch of 4 s, according to the criteria previously reported (23, 32). All phases were manually rechecked and corrected.

### Immunohistochemistry

The mice were anesthetized and perfused with 30 ml of 0.1 M phosphate buffer (PBS, PH 7.0) and 30 ml of ice-cold 4% paraformaldehyde *via* the heart for cervical dissection, and the brains were removed and placed in 4% paraformaldehyde overnight at 4°C. The mouse brains were then transferred to 20% sucrose and stored at 4°C until sunken. Thereafter, they were cut on the coronal surface at a thickness of 30 μm using a frozen sectioning machine (CM1950; Leica, Germany). Immunohistochemistry was performed as described previously (23). Briefly, brain slices were incubated with 0.3% H_2_O_2_ for 30 min, rinsed with PBS, and then incubated with primary antibody for c-Fos (1:1000, Abcam, Cambridge, MA, USA) overnight at 4°C. The next day, they were washed 6 times with 0.01 M PBS and incubated with secondary antibody (1,1,000, Jackson Immunoresearch Inc., West Grove, PA, USA) for 90 min at room temperature. After washing 6 times with PBS, all brain slices were incubated with the avidin-biotin complex for 60 min. The sections were then subjected to peroxidase reaction with 3,3-diaminobenzidine and nickel, and the positive cells were stained black. After washing with PBS, the sections were mounted, dried, dehydrated, and cover slipped. Images were taken using a microscope (ECLIPSE Ni-E, Nikon, Japan).

### Statistical analysis

All data are expressed as mean ± SEM. Multi-factor ANOVA comparing the changes in the amount of time at each stage and EEG power density in the baseline, RS, and PRS conditions. The correlation between the sleep time and anxiety levels were evaluated by Pearson correlation during PRS condition. Student^’^s *t*-test was used to compare changes in anxiety-like behavior over time and c-Fos expression between groups. Statistical significance was achieved at a value of p of 0.05 for all tests.

## Results

### Restraint stress induces anxiety-like behavior in mice

**Figure 1:**
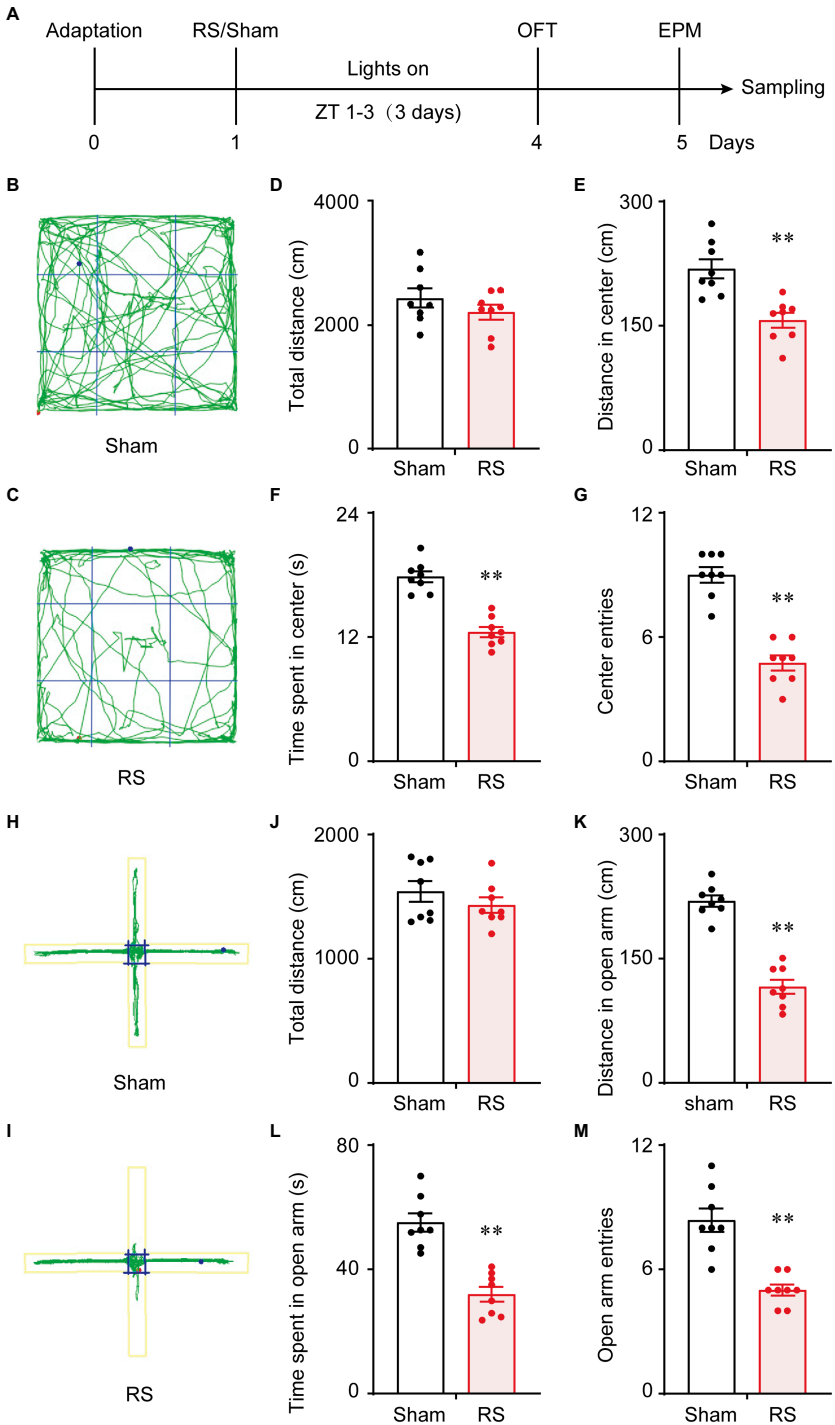
Behavioral experimental schedule and acute restraint stress induced anxiety-like behavior. Schedule for conducting the OFT and EPM **(A)**. OFT trajectory diagram of the sham group **(B)** and the RS group **(C)**. Total distance traveled for 5 min in the OFT, the distance and time spent in the center, and the number of times the mice entered the center of the OFT **(D–G)**. EPM trajectory diagram of the sham group **(H)** and the RS group **(I)**. Total distance traveled during 5 min in the EMP, the distance and the time spent in the open arm, and the number of times the mice entered the open arm of the EPM **(J–M)**. **p < 0.01 vs. sham, *n* = 8. RS, restraint stress; OFT, open field test; EPM, elevated plus maze; ZT, Zeitgeber time.

The behavioral tests of OFT and EPM were used to assess anxiety-like behavior and locomotor activity in mice. The procedure for RS and behavioral testing is shown in Figure 1A. A representative travel path of a mouse in the sham group is shown in Figure 1B. The mouse traversed the center of the maze at a greater frequency than the mouse in the RS group (Figure 1C). However, there was no significant difference in the total distance traveled between the two groups of mice (*t* = 1.589, *p* = 0.156, Figure 1D). The traveled distance (*t* = 5.04, *p* < 0.01, Figure 1E) and time spent in the central area (*t* = 8.86, *p* < 0.01, Figure 1F) was significantly decreased, and the number of entries into the central area was less in the RS group of mice (*t* = 6.561, *p* < 0.01, Figure 1G) compared to the sham controls. Representative traces of one mouse in the sham group in Figure 1H and one mouse from the RS group in Figure 1L in the EPM testing are shown. The mouse in the sham group traveled both in the open and closed arms, whereas the RS mouse traveled more in the closed arms. Similarly, there was no significant difference in the total distance between the two groups in the EPM testing (*t* = 1.103, *p* = 0.306, Figure 1J). The traveled distance (*t* = 9.704, *p* < 0.01, Figure 1K) and time spent in the open arms (*t* = 6.511, *p* < 0.01, Figure 1L) were shorter, and the number of entries into the open arms (*t* = 5.974, *p* < 0.01, Figure 1M) were significantly reduced in the RS group compared to the control group. These results indicate that RS induces anxiety-like behavior independent of the general locomotor ability of mice.

### Restraint stress alters the sleep–wake cycles of mice

**Figure 2:**
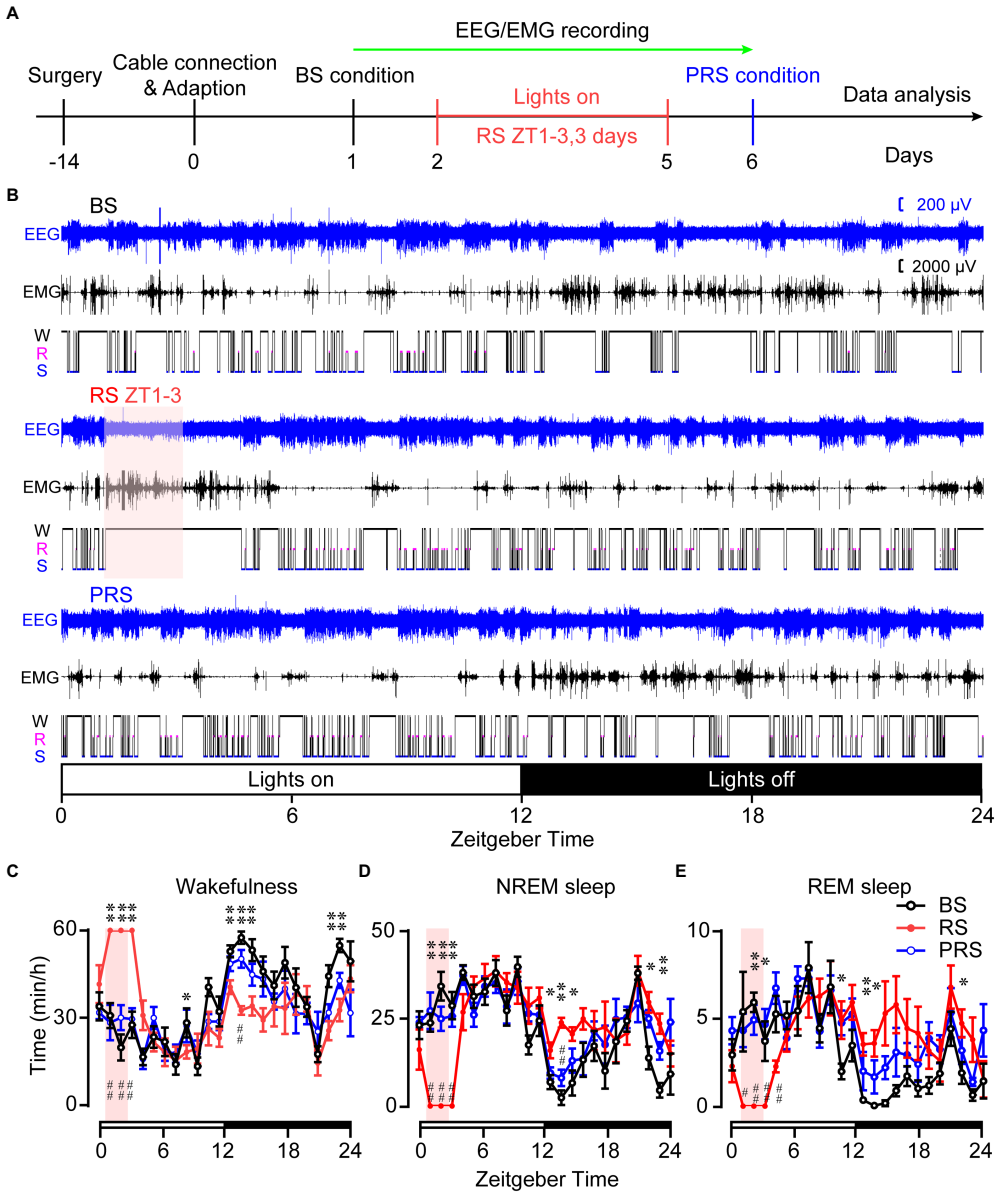
Time spent in the sleep–wake stages of the baseline, RS, and PRS conditions. Timeline and manipulations for conducting the sleep–wake recording. **(A)** Example of 24 h EEG/EMG traces and the corresponding hypnogram under the BS, RS, and PRS conditions. **(B)** Time spent in wakefulness **(C)**, NREM sleep **(D)**, and REM sleep **(E)** during a 24-h period. The pink box indicates restraint stress during 9:00–11:00. The open and closed bars above the *X*-axis indicate the light and dark periods, respectively. *p < 0.05, **p < 0.01 versus baseline, ^#^*p* < 0.05, ^##^*p* < 0.01 versus RS, *n* = 6. BS: baseline; RS: restraint stress; PRS: post-restraint stress; ZT: Zeitgeber time.

**Figure 3:**
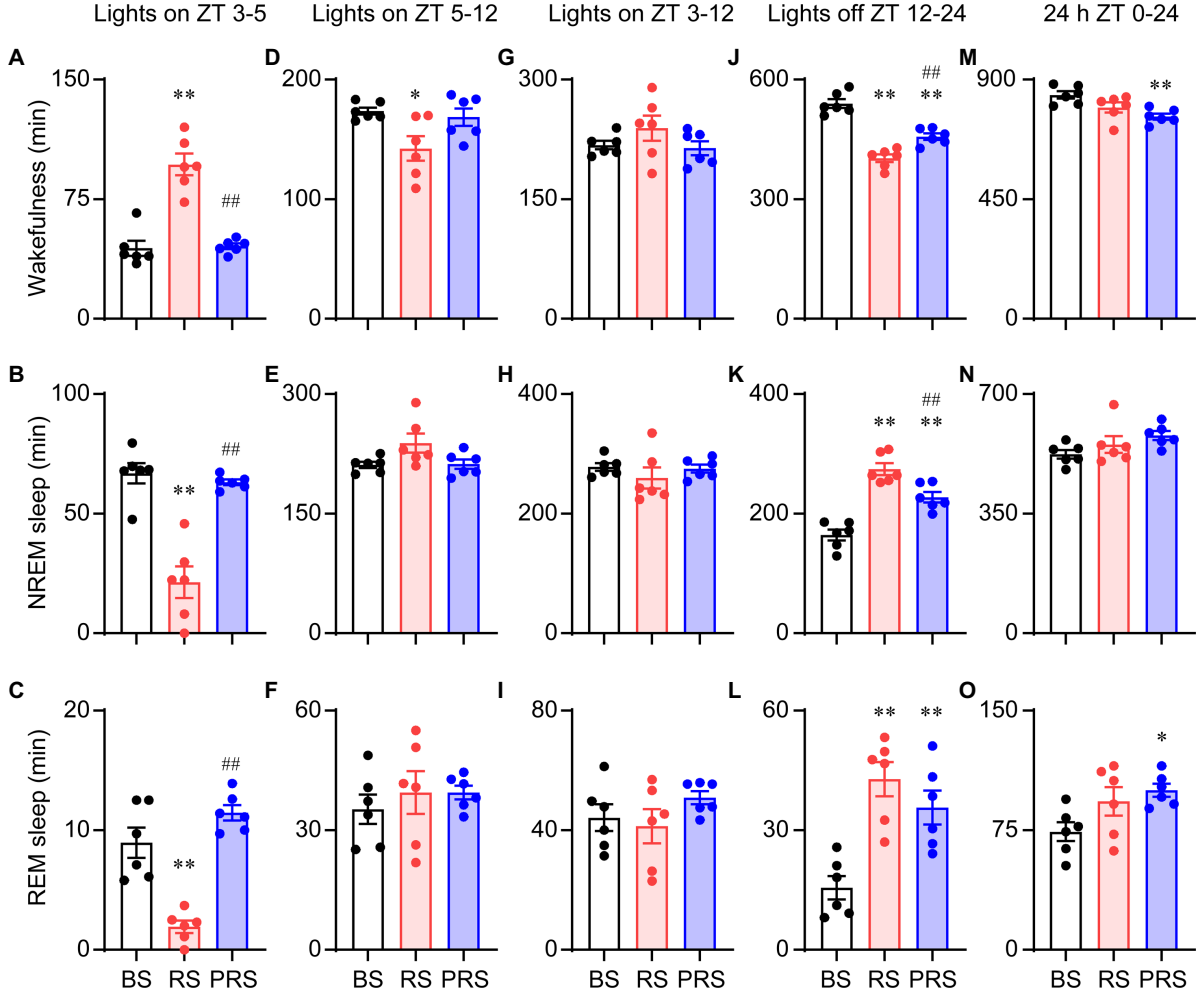
Cumulative time in the sleep–wake stages of the baseline, RS, and PRS conditions. Cumulative time of wakefulness **(A)**, NREM sleep **(B)**, and REM sleep **(C)** during ZT 3–5 (lights on). Cumulative time of wakefulness **(D)**, NREM sleep **(E)**, and REM sleep **(F)** during ZT 5–12 (lights on). Cumulative time of wakefulness **(G)**, NREM sleep **(H)**, and REM sleep **(I)** during ZT 3–12 (lights on). Cumulative time of wakefulness **(J)**, NREM sleep **(K)**, and REM sleep **(L)** during ZT 12–24 (lights off). Cumulative time of wakefulness **(M)**, NREM sleep **(N)**, and REM sleep **(O)** during ZT 0–24 (24 h). *p < 0.05, **p < 0.01 versus baseline, ^##^*p* < 0.01 versus RS, *n* = 6. BS, baseline; RS, restraint stress; PRS, post-restraint stress; ZT, Zeitgeber time.

Next, we assessed the effect of RS on sleep–wake profiles in mice. After capturing the baseline of sleep–wake rhythms, RS was carried out for 2 h (ZT 1–3) in the mice with the sleep–wake recording simultaneously for 3 days, as shown in Figure 2A. Representative traces of the EEG/EMG and hypnogram are shown in Figure 2B. The RS manipulation induced a significantly increased wakefulness and decreased NREM and REM sleep during the manipulation (*p < 0.05, **p < 0.01, Figures 2C–E) when compared to the baseline. After the RS for 3 days, the mice still tended to show an increase in both NREM and REM sleep amount, with decreased wakefulness during the dark period, even under the drive of circadian rhythm and feeding (#*p* < 0.05, ##*p* < 0.01, compared to RS, Figures 2C–E). Furthermore, the cumulated time was calculated during the light and dark periods. During ZT 3–5 (lights on), the time spent in arousals in the RS mice were significantly increased, when compared to baseline (*F*_(2,15)_ = 38.3, *p* < 0.01, Figure 3A). After the 3 days of RS, the amount of wakefulness was returned to the baseline (*F*_(2,15)_ = 38.3, *p* < 0.01, compared to RS). Correspondingly, both time spent in NREM (*F*_(2,15)_ = 30.4, *p* < 0.01, Figure 3B) and REM sleep (*F*_(2,15)_ = 32.3, *p* < 0.01, Figure 3C) were significantly decreased in RS mice compared to the baseline, and the time spent in NREM (*F*_(2,15)_ = 30.4, *p* < 0.01, compared to RS, Figure 3B) and REM sleep (*F*_(2,15)_ = 32.3, *p* < 0.01, compared to RS, Figure 3C) were recovered during the PRS condition. During the ZT 5–12 light period, the time of wakefulness in the RS mice was significantly decreased when compared to baseline (*F*_(2,15)_ = 5.1, *p* < 0.05, Figure 3D). No significant differences were found among the time spent on NREM, and REM sleep in the baseline, RS, and PRS conditions (Figures 3E,F). No significant differences were detected among the cumulative time of wakefulness, NREM, and REM sleep in the baseline, RS, and PRS conditions at ZT 3–12 during the light period (Figures 3G–I). By contrast, during the 12 h dark phase (ZT 12–24), the time spent in wakefulness was less in the RS and PRS states than that in the baseline condition (*F*_(2,15)_ = 50.5, *p* < 0.01, Figure 3J), although the wake time spent in the PRS state was significantly increased compared to the RS state (*F*_(2,15)_ = 50.5, *p* < 0.01, Figure 3J). Conversely, when compared to the baseline condition, both the time spent in NREM (*F*_(2,15)_ = 35.1, *p* < 0.01, Figure 3K) and REM sleep (*F*_(2, 15)_ = 13.2, *p* < 0.01, Figure 3L) in the RS and PRS conditions dramatically increased. When we compared the total amount of wakefulness, NREM sleep and REM sleep during 24 h, the wakefulness time was significantly decreased (*F*_(2,15)_ = 7.3, *p* < 0.01, Figure 3M), whereas the REM sleep time significantly increased (*F*_(2,15)_ = 4.1, *p* < 0.05, Figure 3O) in the PRS condition, compared to baseline. These results indicate that RS manipulation resulted in an initial wakefulness and sleep response during the dark period, even after the termination of RS manipulation.

### Restraint stress changes the sleep–wake architecture of mice

**Figure 4:**
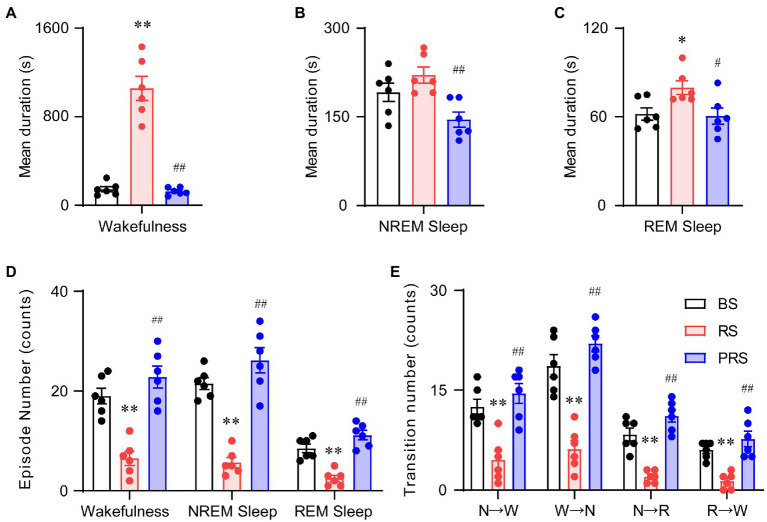
Sleep–wake architectures in the baseline, RS, and PRS conditions during ZT 3–5. Mean durations of wakefulness **(A)**, NREM sleep **(B)**, and REM sleep. **(C)** Episode numbers of each stage **(D)** and stage transitions between stages **(E)** during the 2 h in the lights on period (ZT 3–5) after restraint stress. *p < 0.05, **p < 0.01 versus baseline, ^#^*p* < 0.05, ^##^*p* < 0.01 versus RS, *n* = 6. BS, baseline; RS, restraint stress; PRS, post-restraint stress. R, REM sleep; S, NREM sleep; W, wakefulness.

To assess the sleep–wake architectures of the mice during and after the RS manipulation, we independently calculated the episode number and mean duration of wakefulness, NREM, and REM sleep during the day and night periods. As shown in Figure 4, there was a significant increase in the mean duration of wakefulness in the RS condition at ZT 1–3 (lights on) compared to baseline. A significant decrease in the mean duration of wakefulness was detected in the PRS, compared to RS condition (*F*_(2,15)_ = 66.0, *p* < 0.01, Figure 4A). The mean duration of REM sleep was increased in the RS, compared to baseline (*F*_(2,15)_ = 5.2, *p* < 0.05, Figure 4C). In the PRS condition, both the mean duration of NREM sleep (*F*_(2,15)_ = 7.3, *p* < 0.01, Figure 4B) and REM sleep (*F*_(2,15)_ = 5.2, *p* < 0.05, Figure 4C) were decreased when compared to the RS condition. Correspondingly, the number of wakefulness, NREM sleep, and REM sleep episodes significantly decreased in the RS condition compared to baseline and returned to baseline after the RS manipulation (*F*_(2,15)_ = 41.8, *p* < 0.01, Figure 4D). In addition, the RS manipulation reduced the sleep–wake transitions compared to baseline (*F*_(2,15)_ = 55.7, *p* < 0.01, Figure 4E). Based on these results, the RS resulted in an initial elevated wakefulness by increasing the mean duration and decreasing the number of wakefulness episodes.

**Figure 5:**
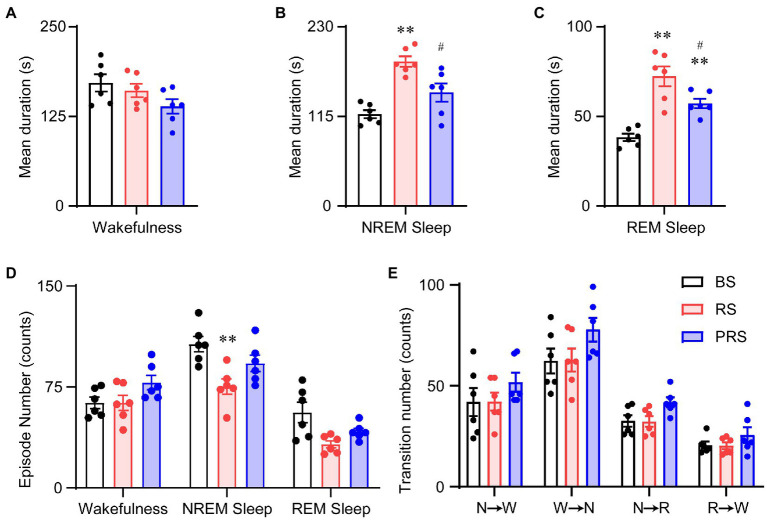
Sleep–wake architectures in the baseline, RS, and PRS groups during ZT 5–12. Mean durations of wakefulness **(A)**, NREM sleep **(B)**, REM sleep **(C)**, and episode numbers for each stage **(D)** during ZT 5–12. **(E)** Stage transitions between stages during ZT 5–12. **p < 0.01 versus baseline, ^#^*p* < 0.05 versus RS, *n* = 6. BS, baseline; RS, restraint stress; PRS, post-restraint stress; R, REM sleep; S, NREM sleep; W, wakefulness.

During the late light period (ZT 5–12), no significant differences were observed in the mean duration of wakefulness among the three conditions (*F*_(2,15)_ = 2.5, *p* = 0.11, Figure 5A). However, the mean duration of both the NREM (*F*_(2,15)_ = 16.5, *p* < 0.01, Figure 5B) and REM sleep (*F*_(2,15)_ = 20.9, *p* < 0.01, Figure 5C) significantly increased in the RS condition, compared with baseline. Compared to the RS condition, the mean duration of REM sleep decreased in the PRS condition, which was still much longer than that in baseline (*F*_(2,15)_ = 20.9, *p* < 0.01, Figure 5C). The episode numbers of wakefulness and REM sleep had no dramatic differences among the three conditions, whereas compared to baseline, the episode number of the NREM sleep was significantly decreased (*F*_(2,15)_ = 6.3, *p* < 0.05, Figure 5D). There were no significant changes in the number of sleep–wake transitions during the three conditions (*F*_(2,15)_ = 3.1, *p* = 0.07, Figure 5E). The above results indicate that RS manipulation resulted in sleep fragmentation, although there were no significant differences between the time spent in NREM and REM sleep.

**Figure 6:**
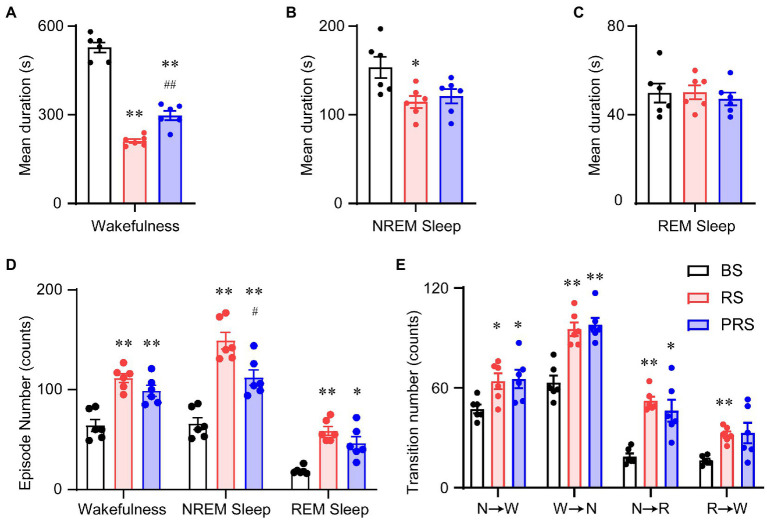
Sleep–wake architectures in the baseline, RS, and PRS groups during ZT 12–24. Mean durations of wakefulness **(A)**, NREM sleep **(B)**, REM sleep **(C)**. Episode numbers of each stage **(D)** during ZT 12–24. **(E)** Stage transitions between stages during 12 h at ZT12–24. *p < 0.05, **p < 0.01 versus baseline, ^#^*p* < 0.05, ^##^*p* < 0.01 versus RS, *n* = 6. BS, baseline; RS, restraint stress; PRS, post-restraint stress; R, REM sleep; S, NREM sleep; W, wakefulness.

In the dark phase at ZT 12–24, the wakefulness duration decreased in both the RS and PRS conditions, compared to the baseline. However, compared to that in the RS, the wakefulness duration in the PRS condition was partially recovered (*F*_(2,15)_ = 135.4, *p* < 0.01, Figure 6A). The NREM sleep duration was reduced in the RS condition (*F*_(2,15)_ = 5.1, *p* < 0.05, Figure 6B). There was no significant difference in the REM sleep duration among the three conditions (*F*_(2,15)_ = 0.2, *p* = 0.8, Figure 6C). In general, the number of episodes of all stages dramatically increased in the RS and PRS conditions at ZT 12–24 compared to the baseline (*F*_(2,15)_ = 37.5, *p* < 0.01, Figure 6D). In addition, there was an increase in the transition numbers between the stages of sleep–wake cycles (*F*_(2,15)_ = 17.5, *p* < 0.01, Figure 6E). These results suggest that the lethargy of mice under RS and PRS conditions during the dark phase depended on the increasing NREM and REM sleep episodes.

### Effect of restraint stress manipulation on sleep power density

**Figure 7:**
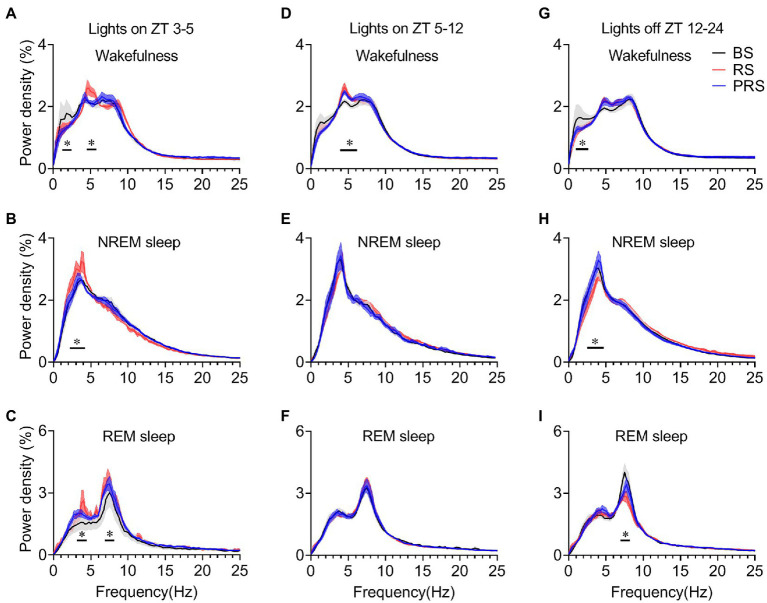
Changes in EEG power density during wakefulness, NREM sleep and REM sleep in the three time periods. EEG power density of wakefulness **(A)**, NREM sleep **(B)** and REM sleep **(C)** during ZT 3–5. EEG power density of wakefulness **(D)**, NREM sleep **(E)** and REM sleep **(F)** during ZT 5–12. EEG power density of wakefulness **(G)**, NREM sleep **(H)**, and REM sleep **(I)** during ZT 12–24. *p < 0.05, compared with baseline, *n* = 6. BS, baseline; RS, restraint stress; PRS, post-restraint stress; ZT, Zeitgeber time.

To assess the effect of RS on sleep quality in mice, EEG power density was calculated among the baseline, RS, and PRS conditions. During the day period at ZT 3–5, the EEG power density of wakefulness decreased of the frequency band from 1.25 Hz to 2.5 Hz, and increased from 4.5 Hz to 5.75 Hz in the RS, compared to the baseline (*p* < 0.05, Figure 7A). Both the frequency band from 2.25 Hz to 4.25 Hz during NREM sleep (*p* < 0.05, Figure 7B) and the frequency band from 3.0 Hz to 4.5 Hz and from 6.75 Hz to 9.25 Hz during REM sleep (*p* < 0.05, Figure 7C) significantly increased in the RS condition compared to the baseline. The EEG power density of wakefulness increased from 4.0 Hz to 5.25 Hz in the RS condition compared to the baseline (*p* < 0.05, Figure 7D). In contrast, the EEG power density of NREM and REM sleep recovered to baseline levels in the PRS condition. During the light period ZT5–12, the difference in EEG power density among the groups was not significant (Figures 7E,F). However, during the dark period (ZT 12–24), the EEG power density of the frequency band from 1.0 Hz to 2.75 Hz of wakefulness, the frequency band from 2.5 Hz to 4.75 Hz of NREM sleep and the frequency band from 7.0 Hz to 8.25 Hz of REM sleep in the RS group decreased significantly compared to the baseline (*p* < 0.05, Figures 7G–I). These results indicate that RS stress affected the sleep quality of the mice in both the light and dark periods.

### Relationship between sleep amount and anxiety levels after RS manipulation

**Figure 8:**
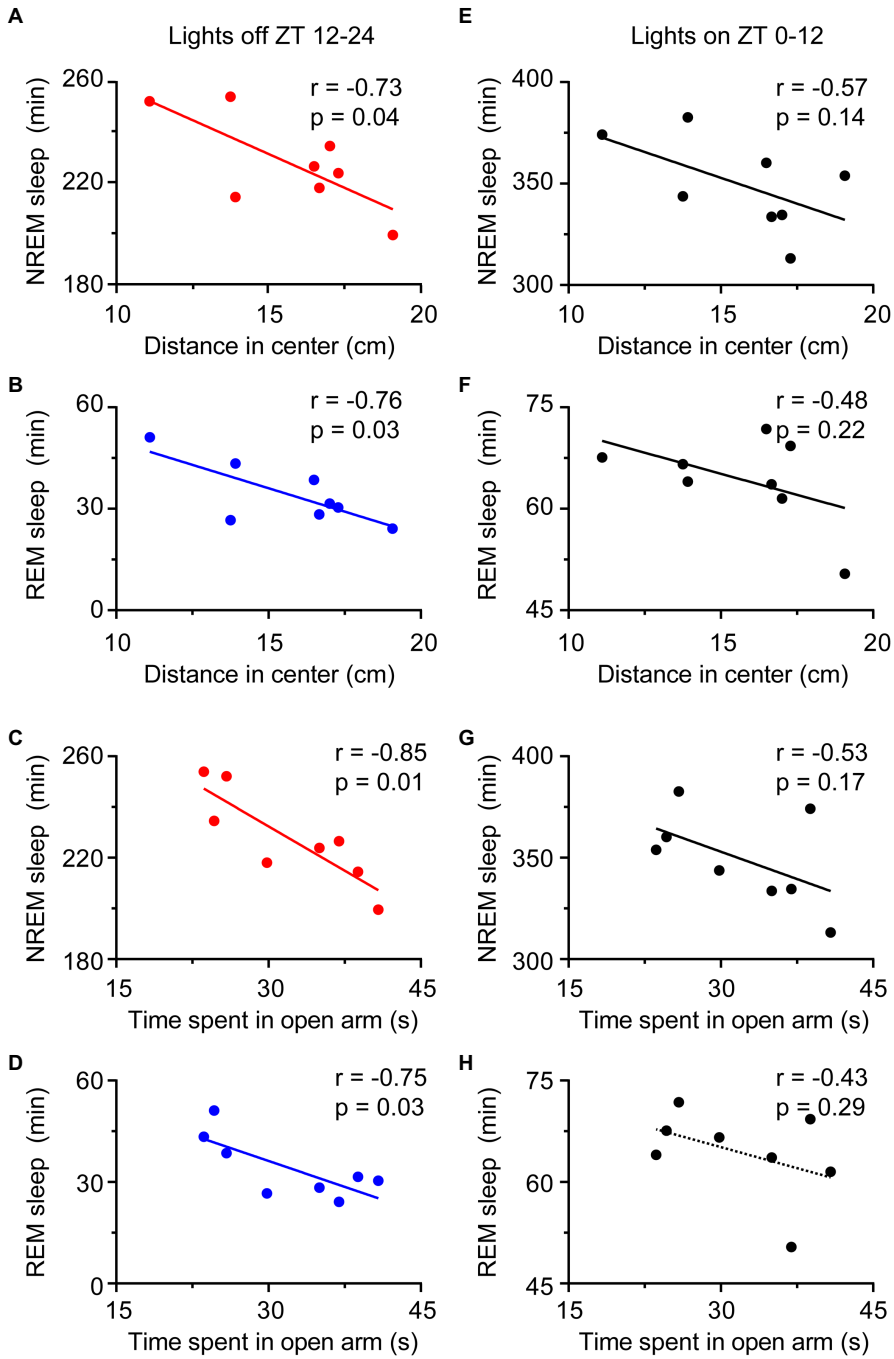
Relationship of sleep amount and anxiety-like behaviors during the PRS condition. The amount of NREM and REM sleep during the night period correlated with anxiety-like behaviors **(A–D)**, but the amount of NREM and REM sleep during the day period was not correlated with anxiety-like behaviors **(E–H)**. *n* = 8. ZT, Zeitgeber time.

Given that anxiety and depression are often preceded by sleep disorders in humans, we next investigated the relationship between sleep amount and anxiety levels after the RS for 3 days (during the PRS condition). The higher anxiety levels of rodents were indicated by the shorter distance traveled in the center arena in the OFT and the less time spent in the open arms of the EPM (33). Both the time of NREM and REM sleep during the dark period was negatively correlated with the distance in the center in OFT and time spent in open arms in the EPM (Figures 8A–D). In other words, sleep time during the night period was positively correlated with anxiety levels after RS treatment. However, there was no correlation between sleep time during the light period and anxiety-like phenotypes (Figures 8E–H). Thus, these results suggest that the increased sleep time during the dark period might reflect anxiety levels after RS manipulation.

### Restraint stress drives the expression of the c-Fos protein in mouse brain

**Figure 9:**
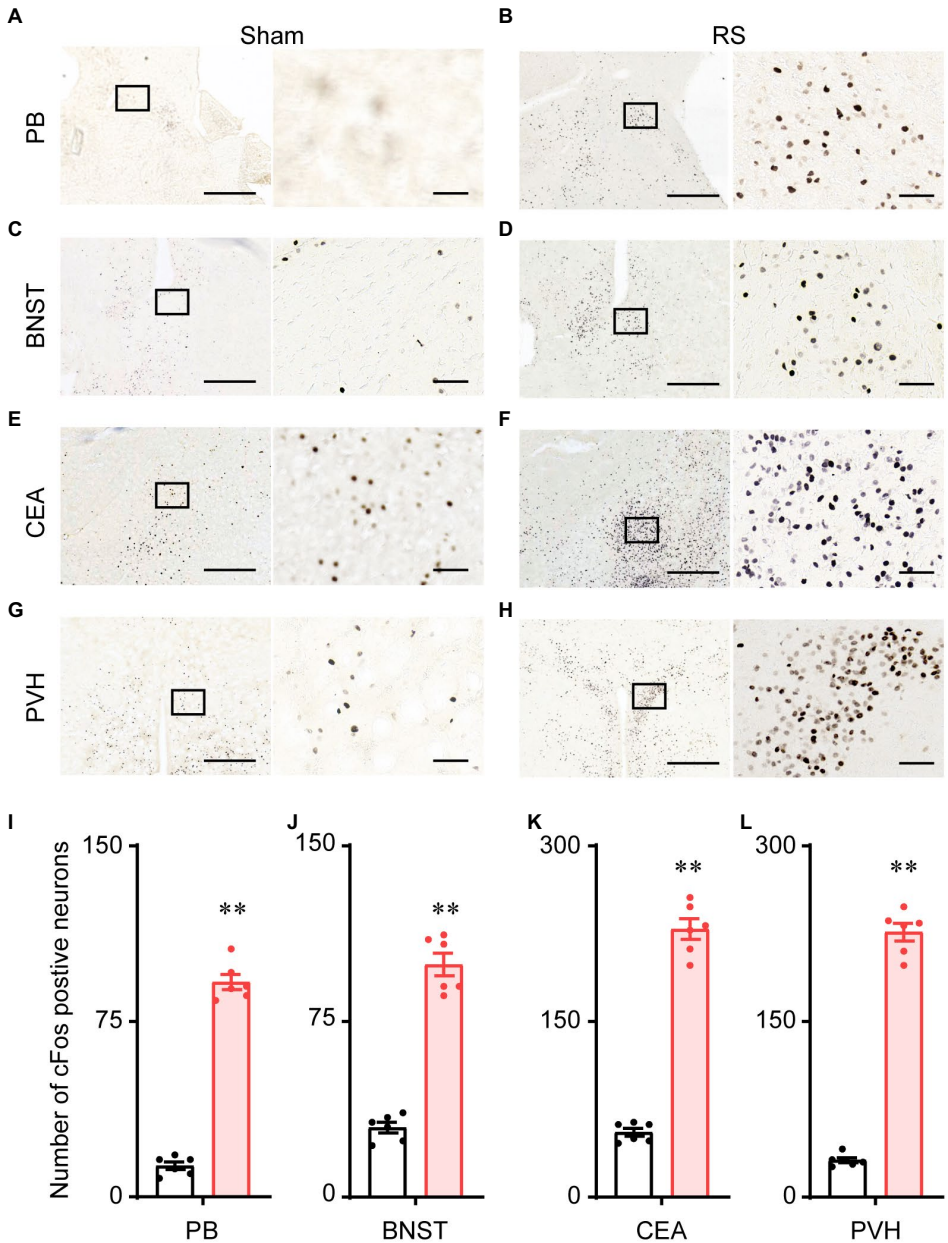
Expression of the c-Fos protein in the mouse brain. Representative images of c-Fos expression in the PB **(A,B)**, BNST **(C,D)**, CEA **(E,F)**, and PVH **(G,H)** after sham and RS manipulation in mice. **(I–L)**. Mean numbers of c-Fos-positive neurons in PB **(I)**, BNST **(J)**, CEA **(K)**, and PVH **(L)**. The right panel shows an enlargement of the box in the left panel. Scale bar: 500 μm in the left panels and 50 μm in the right panels. **p < 0.01, compared with sham, *n* = 6. RS, restraint stress; PB, parabrachial nucleus; BNST, bed nucleus of the stria terminalis; CEA, central amygdala; PVH, paraventricular hypothalamus.

To further investigate the potential mechanism of RS-induced anxiety and sleep, we detected the c-Fos protein by immunohistochemical staining in the brains of the RS mice and sham controls. As shown in Figure 9, very few neurons expressed c-Fos in the parabrachial nucleus (PB, Figure 9A), the bed nucleus of the stria terminalis (BNST, Figure 9C), the central amygdala (CEA, Figure 9E), and the paraventricular hypothalamus (PVH, Figure 9G) in the sham group. In contrast, robust expression of c-Fos was observed in the PB (Figure 9B), BNST (Figure 9D), CEA (Figure 9F), and PVH (Figure 9H) of the RS mice. A quantitative analysis of the number of c-Fos-positive neurons revealed a significant increase in the PB (*t* = 20.5, *p* < 0.01, Figure 9I), BNST (*t* = 11.9, *p* < 0.01, Figure 9J), CEA (*t* = 20.1, *p* < 0.01, Figure 9K), and PVH (*t* = 25.7, *p* < 0.01, Figure 9L) of RS mice, compared to the sham group. These results suggest that the nuclei, including PB, BNST, CEA, and PVH, may contribute to RS-induced changes in sleep with anxiety-like behavior.

## Discussion

In the present study, we performed restraint stress on mice to analyze its effects on anxiety-like behavior and sleep–wake cycles. The results showed that 2 h of RS manipulation for 3 days led to anxiety-like behavior and sleep disturbance. RS also led to a sleep response during the subsequent dark period, when the mice were more active at baseline. In particular, the increased episode numbers of NREM and REM sleep resulted in hypersomnia of RS mice during the dark period, even when the 3 days of RS were terminated. Acute restraint stress-induced sleep fragmentation may affect neural activity in the central nervous system, and thereby contribute to mental disorders. The results of c-Fos expression in the anxiety-and sleep-related nuclei suggested a potential neural network that mediated these effects of restraint stress in mice.

Anxiety disorders are a very common mental illness in the general population (34). Then are the most prevalent class of lifetime psychiatric diseases, with a prevalence of up to 5.0% in China over the last 12 months (5). Here, acute restraint stress for 3 days resulted in anxiety behaviors in mice without leading to impaired mobilization capacity (Figure 1). Numerous studies have shown that acute or chronic restraint stress induces anxiety-like behavior in rodents, which is caused by dysfunction of the amygdala or BNST (13, 14, 35, 36). Similarly, a previous study observed anxiety-like behaviors in mice that underwent immobilization stress with increasing corticosterone levels in the blood and the activation of nuclear factor-κB and microglia in the hippocampus (37). These results indicate that restraint stress comprises both physical stimuli and psychological components that lead to dysfunction of the central nervous system and cause mental disorders. Hospital intensive care units are stressful environments for both patients and their families. When the patients were admitted to the intensive care unit, 32% of their family members experienced anxiety disorders, 16% of family members experienced depression, and 15% of family members experienced posttraumatic stress symptoms during the 3-month follow-up (38). These results suggest that acute or traumatic events can have long-term consequences for the brain and behavior. Therefore, the prevention of acute stress may prohibit such effects from developing into chronic and irreversible pathological states.

Anxiety disorders and depression are accompanied by sleep disturbances (7, 39). Sleep disorders affect more than 50% of patients with anxiety disorders and can lead to increased anxiety (40, 41). Compared to healthy subjects, patients with anxiety disorders showed prolonged sleep latency and a significant reduction in total sleep time and sleep efficiency (42). Here, the mice that suffered from RS showed an immediate loss of both NREM and REM sleep, which was followed by a strong sleep rebound during the dark period (Figures 3J–L), when the mice had to overcome the drive of circadian rhythm and food seeking during the dark period (43). NREM and REM sleep rebounds were still observed during the recovery night, indicating the long-term effect of restraint on sleep in mice. The elevation in NREM and REM sleep times was consistent with previous reports in mice that experienced chronic restraint stress with water immersion (18), or acute social defeat stress (21, 44). Although one of the most characteristic features of sleep in depressive patients is an increase in REM sleep (45, 46), whether increased sleep after restraint is beneficial or detrimental to anxiety is unclear. Our correlation analysis showed that anxiety levels were negatively correlated with sleep time during the post-restraint condition (Figure 8), suggesting that sleep recovery may help relieve anxiety in mice. When people are exposed to trauma, a longer duration of REM sleep episodes is associated with a lower likelihood of developing posttraumatic stress disorder (47, 48). A shorter latency to sleep onset was predictive of lower levels of anxiety-like behaviors in social defeat stressed mice (44). These findings support the idea that sleep plays a beneficial role in treating anxiety. Antidepressants, including serotonin and norepinephrine reuptake inhibitors, monoamine oxidase inhibitors, and selective serotonin reuptake inhibitors, decrease the amount of REM sleep in patients with psychiatric disease (49). REM sleep deprivation or serotonin and noradrenaline re-uptake inhibitors reduced anxiety-like behavior in rodents (50, 51), indicating that the increased REM is detrimental for emotion. In addition, patients with mood disorders often experience sleep fragmentation (52). The mice after restraint showed a decreased mean duration of wakefulness and NREM sleep with increased episode numbers and transitions between each stage (Figures 4–6). These findings demonstrate overnight sleep disruption in restrained mice with anxiety-like behaviors. In mice, a chronic mild stress paradigm can also lead to fragmented sleep, with changes in neurochemistry and neuroendocrine factors (53). Other mechanisms, such as stress-induced corticotropin-releasing hormones and prolactin, may be involved in sleep regulation (20, 54). However, further studies are needed to elucidate the relationship between stress induced sleep and anxiety-like behaviors.

Stress also induces alterations in EEG power density in humans and animals (21, 55). Slow-wave sleep is thought to correlate with changes in the levels of stress hormones (56). Patients with depression often show decreased slow-wave sleep (57). Thus, changes in sleep amount and EEG power density may contribute to the emergence of anxiety and depression-like behaviors in restraint mice. Here, we found that during ZT 3–5 of the light period, the delta power density of NREM sleep increased in RS mice, whereas the delta power density of NREM sleep in PRS conditions returned to the baseline. Similar results were observed in REM sleep across the baseline, RS, and PRS conditions. The increased EEG power density during NREM and REM sleep in RS conditions is consistent with previous reports in mice and rats with acute social defeat stress (21, 58). However, during the dark period, the power density of both the NREM and REM sleep decreased in RS conditions (Figure 7), with the amount of sleep increasing (Figure 3). Our results support the idea that more anxious individuals experience severe sleep disturbances.

The exact neuronal mechanisms of the restraint stress-induced behavior response are unknown. Restraint stress induces anxiety-like behavior with dysfunction of the amygdala (12–14, 35), including connectivity with the medial prefrontal cortex (12) and changes in dendritic spine density and spine type (14). The PB is located in the dorsolateral pons, which plays an essential role in the development of anxiety-related behaviors (59). The PB relays external information, such as pain, itch, and threat to the hypothalamus, CEA, and BNST, which are critical for modulating affective states (60, 61). Consistent with these previous findings, the c-Fos mapping results from the present study showed robust expression of c-Fos in the lateral PB, CEA, and BNST after restraint stress (Figure 9). Yu et al. showed that a subset of neurons in the ventral tegmental area, which project to the lateral hypothalamus, are activated and induce sleep by social defeat. However, the release of corticotrophin-releasing hormone in the PVH can be inhibited by the activation of the same group of neurons in the midbrain (62). The corticotrophin-releasing hormone secreted from the PVH, which initiates the hypothalamic–pituitary adrenal axis, drives corticosterone synthesis and mediates the stress response (63, 64). These results provide insights into the neural mechanisms that link stress and sleep. In addition, the PB plays an important role in sleep–wake regulation (65, 66). The activation of neurotensinergic neurons in the PB induces anxiety-like behavior and increased wakefulness, which largely project to the emotional control nuclei, such as the CEA and BNST (67). Thus, the neural circuits from PB may regulate anxiety-like behavior and sleep responses to restraint stress. Future studies employing cutting-edge technologies, such as optogenetics and chemogenetics may provide more clues to elucidate the neural mechanisms of stress-induced anxiety and sleep.

There has been abundant research confirming that women are more likely than men to have anxiety and mood disorders (68–70). According to a meta-analysis of 29 studies, women are 41% more likely to have insomnia than men. Furthermore, women have a significantly greater risk of sleep disorders than men, especially with increasing age (71). The high prevalence of anxiety and sleep disorders in women may be due to hormonal changes during menstruation, pregnancy, and menopause (72, 73). However, only male mice were used in the present study, which might be a limitation of our study. Thus, we could not compare gender differences in stress-induced anxiety and sleep.

## Conclusion

The present study revealed anxiety-like behaviors and sleep responses related to acute restraint stress in mice. Sleep amount, architecture, and intensity were affected by restraint stress, especially during the dark period. The robust expression of the c-Fos protein in the brain after RS suggests that PB networks may play an important role in coping with stress.

## Data availability statement

The raw data supporting the conclusions of this article will be made available by the authors, without undue reservation.

## Ethics statement

The animal study was reviewed and approved by The Animal Care and Use Committee of Anhui Medical University.

## Author contributions

QX, C-BG, and M-KZ conceived and designed the study. Y-XX, Z-ZJ, Y-YL, and J-LL carried out the experiments. Y-XX, G-YL, Y-LW, and D-XM analyzed the data. C-BG, M-KZ, X-YW, and QX wrote the manuscript. All authors contributed to the study and approved the final version.

## Funding

This study was supported in part by grants-in-aid for scientific research from the National Natural Science Foundation of China (81401100), Natural Science Foundation of Anhui Province (2208085MH225, 2108085MH282, and 1508085QH155), The Key Program in the Youth Elite Support Plan in Universities of Anhui Province (gxyqZD2018020), Natural Science Foundation of Anhui Education Department (KJ2018A0174), Foundational and Clinical Collaborative Research Project from Anhui Medical University (2019xkjT013 and 2022xkjT026), Scientific Research Platform and Base Upgrading Plan of Anhui Medical University (2021xkjT048), the doctoral research fund project of the First Affiliated Hospital of Anhui Medical University (1545), and the National College Students’ Innovation and Entrepreneurship Training Program (202210366039).

## Conflict of interest

The authors declare that the research was conducted in the absence of any commercial or financial relationships that could be construed as a potential conflict of interest.

## Publisher’s note

All claims expressed in this article are solely those of the authors and do not necessarily represent those of their affiliated organizations, or those of the publisher, the editors and the reviewers. Any product that may be evaluated in this article, or claim that may be made by its manufacturer, is not guaranteed or endorsed by the publisher.
